# Vitamin D Insufficiency in Overweight and Obese Children and Adolescents

**DOI:** 10.3389/fendo.2019.00103

**Published:** 2019-03-01

**Authors:** Irina Zakharova, Leonid Klimov, Victoria Kuryaninova, Irina Nikitina, Svetlana Malyavskaya, Svetlana Dolbnya, Anna Kasyanova, Roza Atanesyan, Marina Stoyan, Anastasiya Todieva, Galina Kostrova, Andrey Lebedev

**Affiliations:** ^1^Department of Paediatrics, Russian Medical Academy of Continuous Postgraduate Education, Moscow, Russia; ^2^Department of Paediatrics, Stavropol State Medical University, Stavropol, Russia; ^3^Department of Paediatrics, Almazov National Medical Research Centre, Saint Petersburg, Russia; ^4^Department of Paediatrics, Northern State Medical University, Arkhangelsk, Russia

**Keywords:** child obesity, vitamin D, vitamin D and obesity, vitamin D and obesity in children, adipose tissue and autoimmune inflammation

## Abstract

Excessive body weight and obesity in childhood and adolescence are becoming more and more important unfavorable factors that entail extremely adverse consequences and require close attention of physicians of any specialty. Along with the high prevalence of obesity and metabolic syndrome in pediatric patients, children and adolescents in the majority of countries are diagnosed with vitamin D deficiency. Among the non-calcaemic effects of vitamin D, a significant role is played by its impact on the hormonal regulation of glucose metabolism and the synthesis of adipokines by fat tissue. The review presents literature data indicative of a close pathogenic relationship between vitamin D insufficiency and impaired tissue insulin sensitivity. It demonstrates the role of vitamin D insufficiency in immune reactions resulting in development of subclinical inflammation in fat tissue infiltrated with macrophages and lymphocytes. It also shows the role of adipokines, immune system cells and pro-inflammatory cytokines produced by them in the pathogenesis of obesity, as well as the function of vitamin D as an endocrine and paracrine regulator of the process of inflammation in adipose tissue. The relationships between the principal adipokines (leptin, adiponectin, resistin) are revealed in the presence of normal vitamin D content and in vitamin D deficiency. The carbohydrate and lipid metabolism parameters in overweight children and adolescents with vitamin D insufficiency are analyzed. A high prevalence of vitamin D insufficiency in overweight and obese children and adolescents (increasing along with the severity of obesity) is demonstrated. The review also presents the current recommendations for the correction of vitamin D insufficiency and underlines the need for higher cholecalciferol doses to achieve serum calcifediol targets in overweight and obese children and adolescents.

## Introduction

Prevention of obesity is one of the most important problems of today's medical science, since the rate at which the prevalence of obesity is increasing worldwide indicates a pandemic (1, 2). In 2010, complications related to overweight and obesity resulted in the death of at least 4 million people in the world, in the decrease of the quality of life in 4% of the population every year and 4% of the population become disabled (3). According to WHO data for 2014, 39% of the world's population suffered from excessive weight and 13% from obesity, overweight/obesity afflicted 43 million children under 5 years of age, and this amount is estimated to increase up to 60 million children worldwide by the year 2020 (2). The prevalence of vitamin D deficiency and insufficiency in overweight and obese patients ranges from 5.6% in Canada (4) to 96.0% in Germany (5).

In recent years, there has been a sharp rise in interest in studying the role of vitamin D in the human body. This is due to the fact that there have been accumulated and reappear not only the bone (calcemic) effects of vitamin D, but also completely new effects—non-bone (non-calcemic) (6). According to contemporary views, vitamin D deficiency is associated with an increased risk of diabetes mellitus, arterial hypertension, heart failure, peripheral arterial disease, acute myocardial infarction, various forms of cancer, autoimmune and inflammatory diseases, decreased immune defenses and increased mortality (7). Vitamin D plays an essential role in the regulation of glucose homeostasis, insulin secretion mechanisms, and inflammation associated with obesity (8). Pregnant women, people of color (blacks, Hispanics and anyone with increased skin melanin pigmentation), obese children and adults and children and adults who practice abstinence from direct sun exposure are at especially high risk (9). These studies are the result of understanding that vitamin D is not a vitamin in the classical interpretation. It is a steroidal prehormone with autocrine, paracrine and endocrine action, which through enzymatic processes is consistently transformed into the body into biologically active metabolites that affect various organs and tissues through genomic and non-genomic effects.

## Prevalence of Overweight and Obesity in Children and Adolescents

The diagnosis and definition of obesity in children is challenging. Obesity is not defined by a standard threshold as it is for adults. Instead, measurements are compared with a reference population. Obesity diagnoses in children are usually determined by calculation of body mass index (BMI). BMI values are then plotted on age-and sex-specific growth charts (10). The Centers for Disease Control overweight is most commonly defined at BMI 85-95 percentile and greater than or equal to 95th percentile for obesity (11). The World Health Organization overweight definition 85–97 percentile and obesity greater than or equal to 97 percentile (12).

Four countries that are leaders in the prevalence of childhood obesity in the world: Greece, USA, Italy and Mexico (13). Most overweight and obese children and adolescents live in economically developed countries, this list is topped by the United States. The prevalence of obesity among American children and adolescents soared dramatically between 1970 and 2000 (from 6.5 to 18.0% in children and from 5.4 to 18.4% in adolescents), and now remains at approximately the same level (4). It is currently estimated that 30% of children in North America are overweight or obese (14).

In economically developed Northern European countries (Denmark, Sweden, Norway), the prevalence of obesity in children remains at approximately the same level among natives and is increasing very significantly among immigrants (15).

A steady rise in obesity prevalence among children is currently seen in countries with medium and low income levels. These countries are following the path trod by economically developed countries 40 years ago, as the prevalence of obesity in their pediatric populations is rapidly growing. The leading country in this list is China where the prevalence rates of obesity among girls and boys increased from 0.45 and 0.16%, respectively, in 1985 to 18.16 and 6.58%, respectively, in 2014 (16). In Eastern European countries (Bulgaria, Croatia, Czech Republic, Hungary, Latvia, Lithuania, etc.), the Russian Federation, and Turkey, the prevalence of obesity (including excessive body weight) is in the range of 14.4–19.2% among boys and 11.8–17.6% among girls (17).

## Interrelationship Between Vitamin D and Adipose Tissue

Vitamin D insufficiency and excessive fat accumulation have mutually negative effects as a result of excessive metabolic processes, enzymatic disorders against a background of decreased activity of alpha-hydroxylase, the key enzyme in the biotransformation of calciferol in a fat-infiltrated liver, resulting in accumulation of inactive forms and decreased bioavailability of vitamin D (8, 18).

In obesity, vitamin D affects insulin secretion, tissue sensitivity to insulin, and systemic inflammation. The direct and paracrine effects of vitamin D lead to VDR activation in pancreatic beta-cells, CYP27B1 expression, and local synthesis of 1,25(OH)_2_D (18, 19).

Insulin secretion and tissue insulin sensitivity are Ca^2+^-dependent mechanisms, while vitamin D regulates intracellular concentrations of Ca^2+^ and its passage through the membranes. Additionally, vitamin D positively affects the expression of insulin receptors in peripheral cells and counteracts the systemic immune response by modulating the expression and activity of cytokines (20, 21).

Therefore, the influence of adipose tissue on the metabolism of vitamin D, on the one hand, and its pathogenic role in the obesity development mechanisms, on the other hand, are closely interrelated and represent mutually dependent processes.

Numerous studies have analyzed calcifediol concentrations that may be decreased in obesity. One “superfluous” BMI unit is known to induce a 1.15% reduction in the 25(OH)D concentration (22). In particular, an analysis conducted in 58 obese adolescents demonstrated that a 1% increase in fat weight was associated with a 1.15 ± 0.55 nmol/L reduction in serum calcifediol (23).

There is no consensus as to why calcifediol levels are decreased in obese individuals. The first (and most popular) point of view is that adipose tissue absorbs the fat-soluble vitamin D (24). Some available data reveal that serum 25(OH)D concentrations show a strong inverse correlation with fat volume and a weaker inverse correlation with BMI (22).

Another hypothesis explains the low 25(OH)D concentrations by the fact that obese people lead a sedentary lifestyle and are less active physically, which entails a decrease in exposure to sunlight and in endogenous synthesis of vitamin D (25).

Other interrelated hypotheses appear to be justified too, specifically that vitamin D metabolism and 25(OH)D synthesis are impaired as a result of hepatic steatosis developing in obesity (26), and that high levels of leptin and IL-6 impair 25(OH)D synthesis by affecting VDR receptors (27).

## Adipose Tissue and Adipose Tissue Inflammation

As the body weight grows and the energy balance is positive, the amount of adipose tissue unavoidably increases and its distribution, cell composition, and functions change. An increase in the body's adipose tissue volume results in physiological changes, adipocyte hypertrophy (not hyperplasia), ectopic fat deposition, hypoxia, and chronic stress, which eventually leads to impairment of adipokine secretion. It is adipocyte hypertrophy that plays the key role in the loss of cell insulin sensitivity (28). Hypertrophic adipocytes secrete pro-inflammatory factors (leptin, IL-6, IL-8), as the production of insulin-sensitive adipokines (adiponectin and IL-10) decreases (29).

Adipokines are synthesized by adipocytes and affect carbohydrate and fat metabolism. *In vitro* studies have demonstrated that Ca^2+^ and 1,25(OH)_2_D regulate the expression of adipokines in visceral adipose tissue, thus leading to an assumption that vitamin D has a modulatory effect on the expression of the genes responsible for secretion of leptin and adiponectin. Protein spectrum studies conducted in obese children, either vitamin D-deficient or with no vitamin D insufficiency, revealed a direct effect of calcitriol that raised adiponectin levels, leading to a conclusion that adiponectin is a key messenger in the mutual influences of vitamin D and progressive obesity in children. According to the majority of authors, adipokines (leptin, adiponectin) are important predictors of impaired sensitivity to insulin, which indirectly decreases gluconeogenesis in the liver, augments glucose transport into the muscles, correlates with the vitamin D reduction, and shows an inverse relationship with insulin resistance (29, 30).

Adipokines include adiponectin, leptin, tumor necrosis factor (TNF-alpha), plasminogen activator inhibitor type I, transforming growth factor (TGF) type I, and resistin (30). Adipokines regulate fat homeostasis by influencing appetite (amount of ingested food), lipid and carbohydrate metabolism, vascular remodeling, and insulin sensitivity (30).

## Adipose Tissue and its Effects on Adipose Tissue Inflammation

Adipose tissue is heterogeneous, and contains adipocyte precursors (preadipocytes), nerve endings, blood vessels, and white blood cells. The entire complex is called the “stromal vascular fraction.”

In 2003, Xu et al. (31) demonstrated that obesity is associated with a large amount of macrophages in the stromal vascular fraction of adipose tissue. Macrophage migration occurs as a result of impaired functioning of adipose tissue and elevated free fatty acid concentrations (32), production by adipocytes of the proteins chemoattractant-1 and alpha-4 integrin promoting adhesion of macrophages to the endothelial wall, and their subsequent passage through the endothelial barrier (33). Another chemoattractant, LTB4, promotes accumulation of neutrophils in adipose tissue. It is also produced by adipocytes as a result of excessive energy consumption (34). Macrophages accumulate in the visceral pool of adipose tissue. Macrophages migrating into adipose tissue become differentiated in a direction dependent on the volume of the adipose tissue and consequently on the concentration of adipokines generated in adipose tissue. Fat tissue excess is associated with pathological M1-transformation (differentiation) of macrophages. Classical M1 macrophage transformation develops under the influence of T1-helper cells and interferon-gamma or bacterial byproducts. M1-macrophages are pro-inflammatory factors secreting TNF-alpha and IL-1-beta, they have an enormous phagocytic and bactericidal potential (35). On the contrary, Th_2_-cells secrete IL-4, IL-10, IL-13 and promote macrophage transformation through the M2 pathway. M2-macrophages have antiparasitic effects, promote tissue repair and remodeling, and secrete the anti-inflammatory mediator IL-10 (36). Accumulation of macrophages in adipose tissue and their inflammatory activity, along with altered balance of pro- and anti-inflammatory cytokines, is a key element in the pathogenesis of diabetes mellitus type 2, cerebrovascular disorders, and non-alcoholic fatty liver disease in patients with obesity (32, 37).

**Figure 1:**
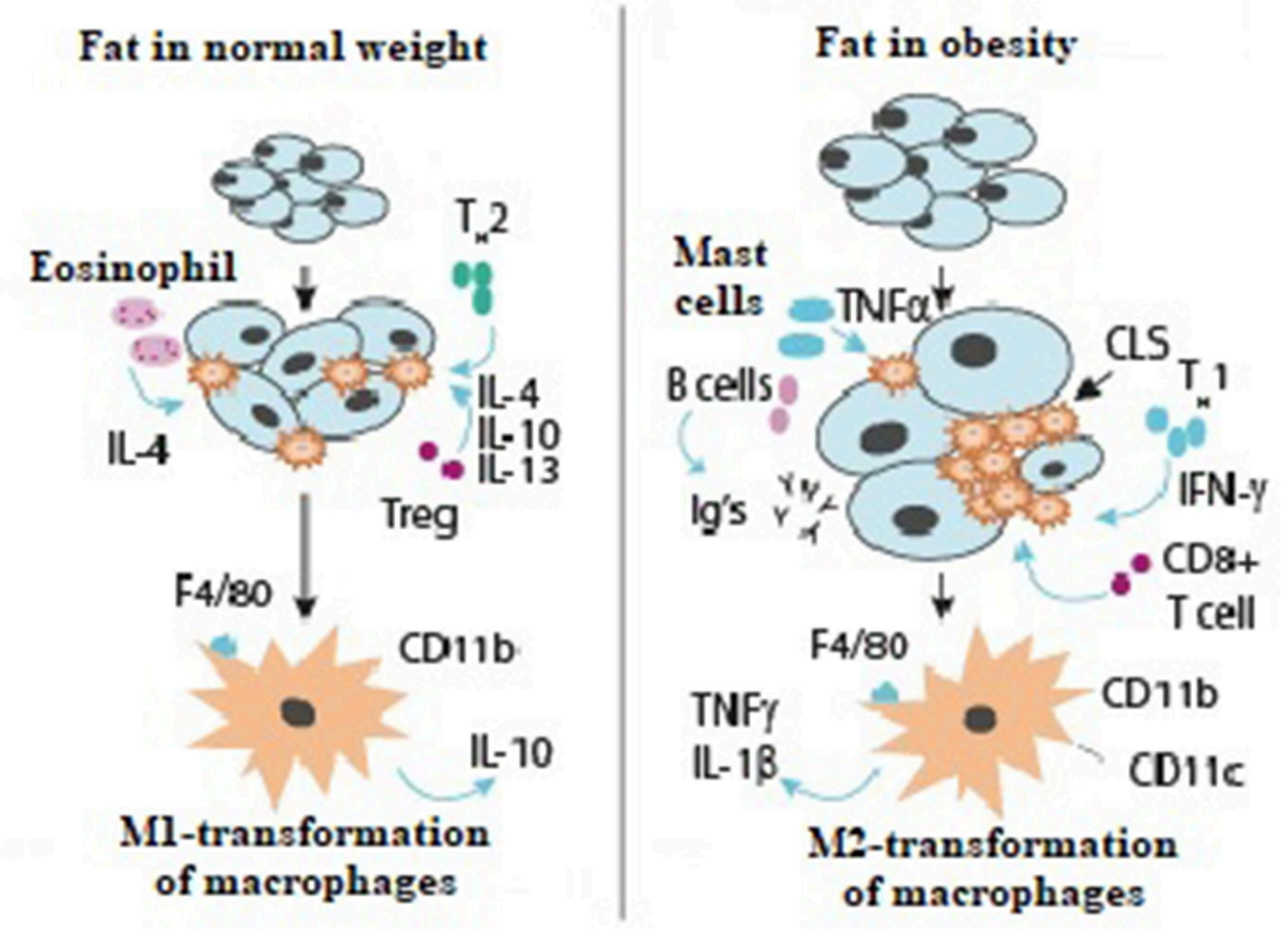
The role of the immune system in healthy adipose tissue and in obesity (37, 38).

The interactions of immune system cells in healthy adipose tissue and in obesity are shown in Figure 1.

Type 2 T-helper cells produce the anti-inflammatory interleukins IL-4, IL-10, and IL-13, which activate M2 macrophage transformation. M2 macrophage transformation is always promoted by T-regulatory cells and eosinophils and mediated by IL-4. M2-macrophages secrete other anti-inflammatory mediators, IL-10, which maintain tissue sensitivity to insulin.

In obesity, Type 1 T-helper cells stimulate M1-macrophage transformation by interferon-gamma; there is also an increased content of other immune cells, B-cells, which synthesize immunoglobulin. As a result, insulin resistance persists. CD8 cells promote macrophage accumulation and augment the expression of pro-inflammatory genes. This results in accumulation of macrophages around dead adipocytes, leading to formation of crown-like structures. M1-transformed pro-inflammatory macrophages secrete TNF-alpha, IL-1-beta, and the marker CD11c.

**Figure 2:**
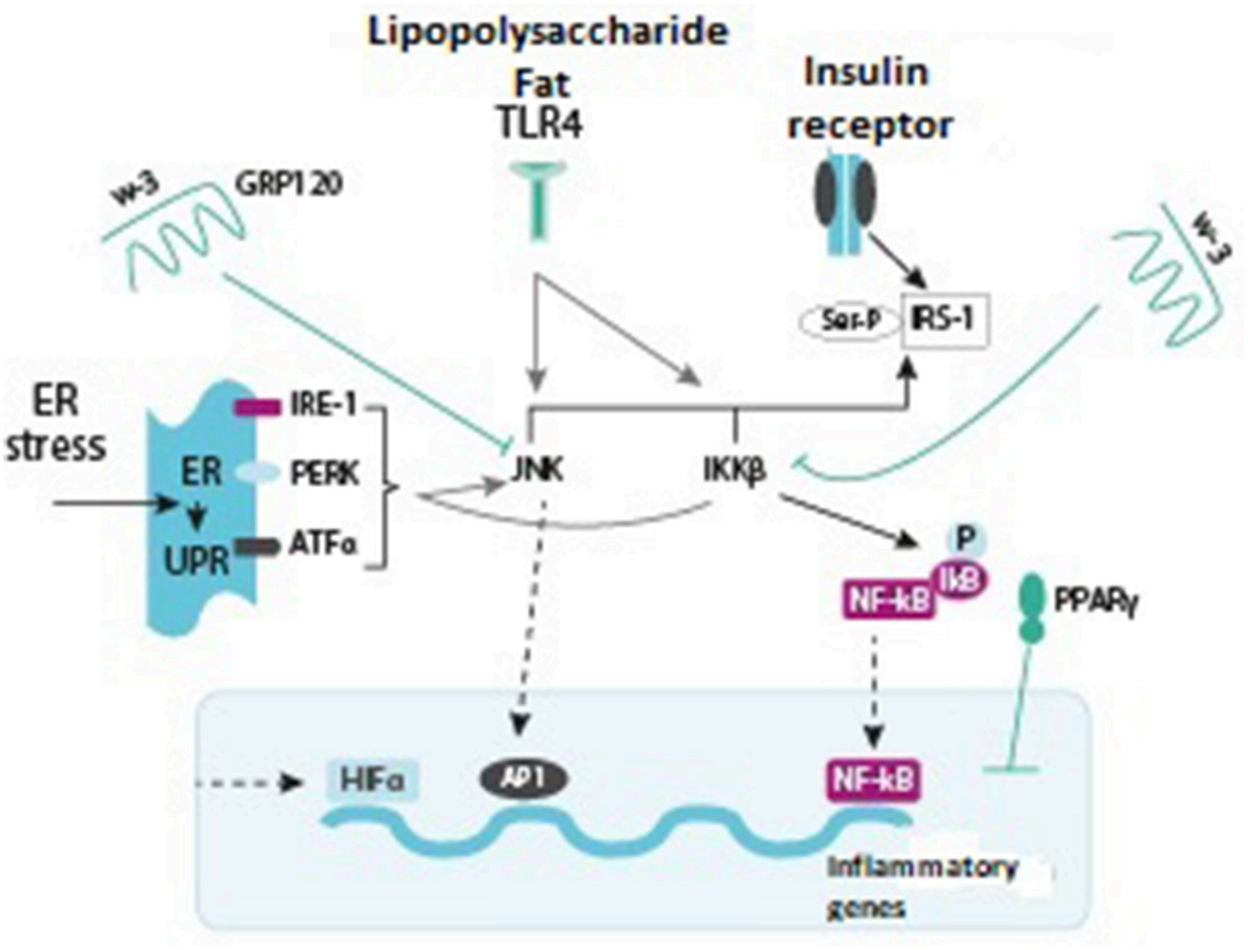
Various signaling pathways stimulating or inhibiting inflammatory signals (green arrows indicate activation, red arrows show inhibition) (36, 37).

Obesity-associated insulin resistance is accompanied by elevated levels of pro-inflammatory cytokines, such as TNF-alpha, IL-6, and IL-1-beta (36). Pro-inflammatory cytokines activate intracellular inflammatory pathways, which results in activation of Jan N-terminal kinase−1 (JNK1) and inhibition of kappa-B kinase-beta (IKK-beta). Products of intracellular cytokine activation decrease insulin sensitivity of the receptors, thus triggering the development of insulin resistance. The kinase activation in obesity demonstrates how closely interrelated metabolic and immune processes in adipose tissue are. Characteristically, JNK1 and IKK-beta are the kinases activated by inherited immune response mediated by Toll-like receptors (TLR) that can be stimulated by lipopolysaccharides, peptidoglycans, double-stranded RNA, and other microbial components (Figure 2) (36).

Jan N-terminal kinase-1 (JNK1) and inhibitor of nuclear factor kappa-B kinase subunit beta (IKK-beta) are inflammatory signaling pathways that promote insulin resistance. Activation of any of the pathways results in phosphorylation of serine, the protein forming a subunit of the insulin 1 receptor, thus counteracting the effects of insulin. IKK-beta also phosphorylates inhibitor of nuclear factor kappa-B (NF-kB), allowing the latter to translocate into the nucleus, bind to the DNA, and activate inflammatory mediators. JNK1 is also able of stimulating the transcription of inflammatory genes in combination with protein transcription activation factor-1 (AP-1). Toll-like receptor-4 (TLR 4), which usually binds lipopolysaccharides (LPS) and saturated fatty acids (FA), promotes activation of JNK1 and IKK-beta. The endoplasmic reticulum (ER) and stress stimulate FA. Excess of nutrients and micro-hypoxia lead to unfolded protein response (UPR). UPR consists of three main pathways: inositol-requiring enzyme (IRE)-1, protein kinase RNA-like endoplasmic reticulum kinase (PERK), and activating transcription factor (ATF) alpha, all of them leading to activation of JNK1 and IKK-beta. Hypoxia also activates a transcription factor, hypoxia-inducible factor-1-alpha (HIF-1α), which induces the expression of different target genes. On the other hand, insulin sensitivity promotes activation of the omega-3 fatty acid receptor (GRP120), which inhibits JNK1 and IKK-beta. PPAR-gamma also augments insulin sensitivity by affecting the NF-kB and AP-1 factors and the subsequent expression of inflammatory genes (36).

**Figure 3:**
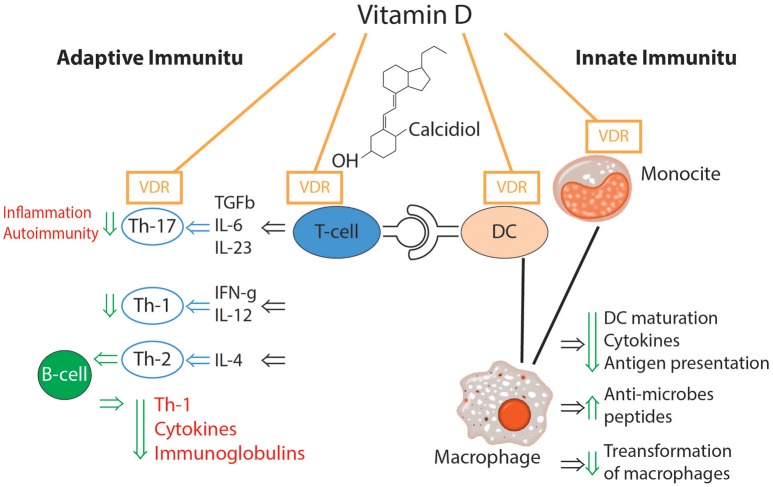
Effects of vitamin D on congenital and acquired immune response (39–42).

The effects of vitamin D on the immune system are multiple and versatile (39). The impact of vitamin D on different elements of immune-mediated inflammation is presented in Figure 3 (39–42).

## Mechanism of Action of Vitamin D in Obesity

Vitamin D that was synthesized in the skin or ingested with food enters the systemic circulation and undergoes 2 stages of hydroxylation. The first stage takes place in the liver forming 25(OH)D3, the second stage takes place in the kidneys forming 1,25 (OH) 2D. This active metabolite provides the basic classical (calcemic) effects, phosphorus-calcium metabolism with parathyroid hormone, and when interacting with the VDR (Vitamin D receptor) receptors in the tissues—non-calcemic effects (43). The active metabolite of vitamin D affects the kidneys, regulating the renin–angiotensin–aldosterone system (RAAS), modulates congenital and acquired immunity, exerts effects on adipose tissue and pancreatic beta-cells, alters insulin sensitivity of the cells, and improves the lipid profile. As a result of its influence on the pancreas, and the beta-cells in particular, expression of insulin receptors is increased and insulin sensitivity is augmented. In adipose tissue, vitamin D counteracts gluconeogenesis, raises HDL cholesterol concentrations, promotes changes in the adipokine profile, and increases leptin levels. Vitamin D has an important non-calcaemic effect, modifying the risk of diabetes mellitus type 2 and altering the adipokine secretion profile, while not decreasing and not affecting body weight (44). Insulin secretion depends on a number of factors, including calcium (45). Vitamin D affects the function of the protein calbindin and acts as a modulator of depolarization-stimulated insulin release by re-distributing intracellular calcium (46). Vitamin D has an effect on insulin sensitivity through a number of mechanisms: by stimulating the expression of insulin sensitivity genes (19, 47), by interacting with the VDR-receptor located in the cell nucleus. The result is an increase in the transcriptional activity of the insulin receptor gene increasing the total number of insulin receptors while not changing their affinity (48). 1,25(OH)_2_D can also augment insulin sensitivity by activating peroxisome proliferator-activated receptor delta (45). Vitamin D insufficiency also leads to elevated parathyroid hormone concentrations, decreased insulin sensitivity, activated lipogenesis, and an increase in fat mass (49). Vitamin D indirectly affects insulin resistance through the RAAS (50).

1,25(OH)_2_D after binding the receptor forms a heterodimer with retinoid X receptor (RXR) and translocates to the nucleus, where this complex interacts with specific DNA regions, called vitamin D-responsive elements. By additional interactions with coregulatory proteins, the VDR–RXR complex regulates approximately 3 percent of the human genome (51).

VDR as a member of steroidhormone receptor super family, it has an essential role in modulating immune response and inflammation via binding with its counter ligand vitamin D. The complex of vitamin D and its receptor controls the B-cell insulin secretion (52, 53). VDR is reported to be expressed in human subcutaneous adipose tissue and visceral adipose tissue (54) and human mammary adipocytes (55). VDR are widely distributed along several body tissues, their gene polymorphisms may affect the risk of vitamin D-related metabolic disorders, and could adjust the receptor effectiveness according to vitamin D status (56, 57). Primarily 4 VDR polymorphisms, including the rs10735810 FoKI SNP and 3 additional ones (the rs7975232 ApaI, the rs1544410 BsmI, and the rs731236 TaqI), have been analyzed in relation to genetic predisposition to obesity; however, findings are contradictory (56). There are now studies that confirm both the positive and negative relationship. The Correa-Rodríguez M study in Spain conducted on Caucasian young adults population (58), Hasan et al. (59) study on the Arab adults residing in the United Arab Emirates; Rahmadhani R in Malaysia (60), did not demonstrate a reliable association with obesity, but found association with the metabolic syndrome components, reduced vitamin D levels, insulin resistance (60), and Fokl and Bsml—with systolic blood pressure (59). Ruiz-Ojeda in the 1,020 review draws the following conclusions: obesity reported that associations with VDR polymorphisms could be related to either a direct effect of vitamin D in adipocyte differentiation and metabolism, or an indirect effect by modulation of insulin secretion (56).

The effect of vitamin D on inflammation in obesity is made up of the following components: *In vitro*, 1,25(OH)2D inhibits chronic inflammation resulting from obesity, the active metabolite of vitamin D 1,25 (OH) 2D inhibits the pro-inflammatory cytokines IL-1β, IL-6, IL-8, IL-12 (39–42, 44, 61), reduces inflammatory activity in adipocytes (62) and reduces inflammation in visceral adipose tissue, while not reducing in subcutaneous fat tissue (62, 63). In obese people a reduced adenosine monophosphate-activated protein kinase and it is closely associated with adipose tissue inflammation. Adenosine monophosphate-activated protein kinase enhances sirtuin 1 by increasing NAD/NADH ratio and decreases adipose tissue macrophage infiltration and inflammation, both have been proposed as key regulators to prevent obesity and obesity-related metabolic dysfunction (64). A 5-year observational study in overweight and obese patients revealed decreased TNF-alpha levels in individuals with normal vitamin D content, as well as a reduction of adipose tissue inflammation (65). In turn, TNF-α regulates the activity of three miRs (miR-146a, miR-150, and miR-155) in adipocytes (66). Vitamin D exerts anti-inflammatory effects mediated by the inhibition of the NF-κB and mitogen activated protein kinase signaling pathways (66), reduced toll-like receptor expression (67). The latter are transmembrane proteins that trigger classical cascade reactions leading to the activation of TNF-alpha (68). The active vitamin D metabolite has an effect on the regulation of NF-kB, the principal transcriptional factor for TNF-alpha; it also blocks the differentiation of dendritic cells and inhibits lymphocyte proliferation (69). *In vitro*, 1,25(OH)_2_D regulates the differentiation of macrophages, suppresses IL-6, and increases the level of the mRNA factor that affects macrophage transformation (70).

Exerting an immunoregulatory effect, vitamin D helps modulate immune response in adipocytes (54) by changing the concentrations and secretion profiles of adipokines (71), inhibits adiponectin synthesis (72), and increases leptin synthesis (73). The NHANES III trial (74) demonstrated that serum calcidiol levels above 21 ng/mL help reduce the C-reactive protein concentration. Vitamin D counteracts the systemic inflammation effect in patients with type 2 diabetes through a number of mechanisms. 1,25(OH)_2_D protects pancreatic beta-cells from cytokine-induced apoptosis, affecting the expression and activity of the cytokines (69). The mutual effects of vitamin D insufficiency and obesity are specific in that, apart from abnormal glucose regulation parameters (75, 76), increased HOMA-index values, dyslipidaemia (77, 78), and elevated systolic blood pressure (77), afflicted children are at increased risk for developing atherosclerosis at an early age (79).

A study that was conducted in the Russian Federation by a group of investigators headed by I. L. Nikitina and evaluated the effects of vitamin D in overweight and obese patients yielded data indicating that vitamin D insufficiency in such children aggravates insulin resistance and promotes lipid profile disturbances (80).

One of the latest meta-analyses that examined the association between vitamin D supplementation and systemic inflammation in patients with diabetes mellitus type 2 demonstrated that dietary cholecalciferol supplementation helps achieve a significant reduction in the activity of inflammation, and also confirmed the data showing that sufficient vitamin D levels help decrease C-reactive protein and TNF-alpha concentrations, decrease ESR, leptin concentrations (73).

**Figure 4:**
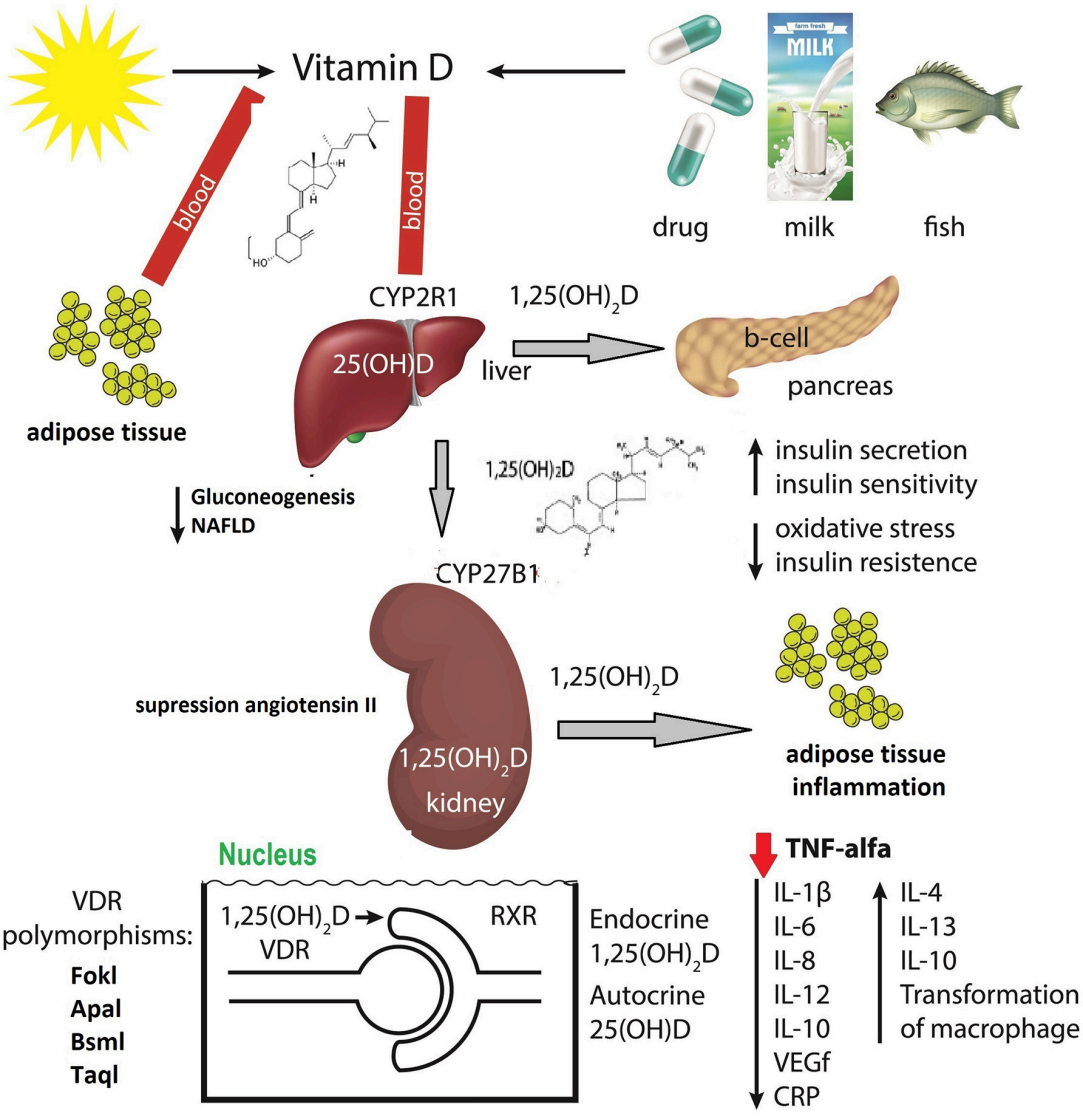
The mechanism of action of vitamin D in obesity (44, 81–83).

Figure 4 demonstrates the mechanism of action of vitamin D on different pathogenic elements in obesity (44, 81–83).

## Prevalence of Vitamin D Insufficiency in Overweight and Obese Children and Adolescents

The association between vitamin D insufficiency and obesity has been extensively investigated in adults. The largest meta-analysis performed by Chinese investigators in 2015 demonstrated a high risk of developing vitamin D insufficiency (84).

**Figure 5:**
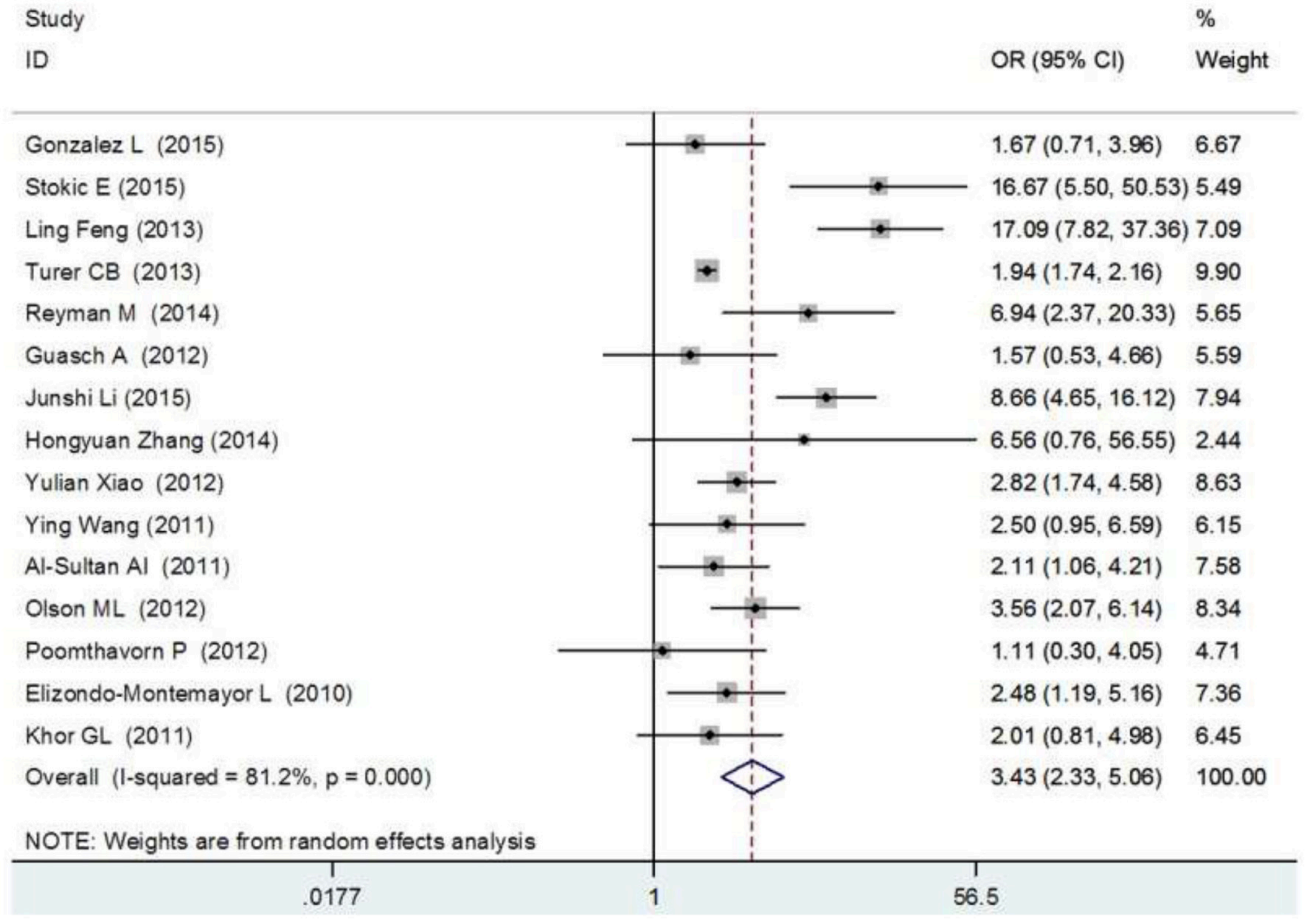
Forest plot between vitamin D deficiency and obesity (84).

The meta-analysis, which included 15 studies (3,867 obese individuals and 9,342 healthy subjects), demonstrated a pronounced difference in vitamin D insufficiency prevalence among obese patients, the OR (95%) was 3.70 (2.33–5.06) (Figure 5).

The prevalence of vitamin D insufficiency in overweight and obese children and adolescents has been investigated in rather good detail, but no dedicated meta-analyses have been carried out. Table 1 summarizes the prevalence of vitamin D insufficiency in groups of overweight and obese children by region and vitamin D sufficiency or insufficiency.

**Table 1:** Prevalence of vitamin D insufficiency in groups of overweight and obese children by region and vitamin D sufficiency or insufficiency.

	**Vitamin D insufficiency level**	**Prevalence of vitamin D insufficiency among overweight/obese children**	**Reference number**
**AMERICA**			
Canada	<50 nmol/L (<20 ng/mL)	5.6% (77.0%–consumption of vitamin D-fortified milk)	(85)
Canada	<75 nmol/L (<30 ng/mL)	93.0%	(86)
Canada	<75 nmol/L (<30 ng/mL)	76.0%	(87)
Mexico	<75 nmol/L (<30 ng/mL)	36.0%	(88)
USA, *New York*	<50 nmol/L (<20 ng/mL)	55.0%	(89)
USA, *Brooklyn*	<50 nmol/L (<20 ng/mL)	55.2%	(90)
USA, *Alabama*	<50 nmol/L (<20 ng/mL)	78.4%	(91)
USA, Pennsylvania	<75 nmol/L (<30 ng/mL)	27.8% (5–9 years) 35.4% (10–14 years) 50.9% (↑ 15 years)	(92)
USA, *Wisconsin*	<50 nmol/L (<20 ng/mL)	32.3%	(93)
**AFRICA**			
Ethiopia	<50 nmol/L (<20 ng/mL)	42.0%	(94)
**EUROPE**			
Denmark	*Deficiency* <30 nmol/L (<12 ng/mL)	16.5%	(95)
Germany	<75 nmol/L (<30 ng/mL)	96.0%	(5)
Greece	<50 nmol/L (<20 ng/mL)	obesity−60.5% overweight−51.6	(96)
Norway	<75 nmol/L (<30 ng/mL)	50.0%	(97)
Spain	<75 nmol/L (<30 ng/mL)	morbid obesity−81.1% obesity−68.2% overweight−55.0%	(98)
Sweden	<50 nmol/L (<20 ng/mL)	33.2%	(99)
The Netherlands	<50 nmol/L (<20 ng/mL)	24.5%	(100)
The Russian Federation, *Arkhangelsk*	<75 nmol/L (<30 ng/mL)	90.0%	(101)
The Russian Federation, *Saint Petersburg*	<75 nmol/L (<30 ng/mL)	92.0%	(102)
**ASIA**			
Iran	<75 nmol/L (<30 ng/mL)	95.6%	(103)
Malaysia	<50 nmol/L (<20 ng/mL)	obesity−19.2% overweight−17.4%	(104)
Turkey	25–50 nmol/L (<20 ng/mL)	23.0%	(105)
China	<75 nmol/L (<30 ng/mL)	48,6%	(106)

Analyzing the data presented in Table 1, one can conclude that the prevalence of vitamin D insufficiency in groups of overweight and obese children is very high. The prevalence of vitamin D insufficiency will be even higher if the vitamin D insufficiency threshold is raised to 75 nmol/L (30 ng/mL), as recommended by the United States National Endocrine Society (107).

This makes northern countries with low sun exposure levels, which are likely to suffer from vitamin D deficiency, fortify foods with vitamin D, as demonstrated in some studies in Canada (85), and recommend prophylactic use of cholecalciferol at high doses and for a long period, as demonstrated in a population of healthy children in Arkhangelsk (The Russian Federation) (108), but overweight children still receive insufficient attention (101).

## Vitamin D Doses in Overweight and Obese Children

There is no conventional dose universally recommended for the treatment of vitamin D insufficiency in overweight and obese children. A number of individual recommendations are available as part of national consensus documents on the treatment of vitamin D insufficiency or included in a number of prospective studies. In particular, the “National programme for vitamin D insufficiency in children and adolescents in the Russian Federation: the state-of-the-art approaches to treatment” recommends determination of the serum calcifediol concentration in children with excessive body weight or obesity, or administration of the maximum prophylactic doses when such measurement is unfeasible (109).

The Committee on Nutrition of the French Society of Pediatrics recommends administration of vitamin D 80,000 IU single doses and 100,000 IU single doses in the winter months (November and February) for obese children aged 5–10 years or uninterrupted supplementation over the age interval of 1–10 years (110).

The United States Endocrine Society, which published its guidelines on the evaluation, treatment, and prevention of vitamin D deficiency in 2011, recommended a twofold increase in the therapeutic dose of cholecalciferol for overweight and obese patients and setting the calcifediol target at 75 nmol/L (30 ng/mL), with subsequent switching to a maintenance dose (107). The recommended therapeutic dose for *healthy* children aged 1–18 years is 2,000 IU/day for 6 weeks or 50,000 IU once weekly for 6 weeks, and the recommended maintenance dose is in the range of 600–1,000 IU/day.

One of the studies enrolled 18 obese adolescents (median BMI: 32.2 kg/m^2^) and 18 non-obese adolescents (median BMI: 20.1 kg/m^2^), who received cholecalciferol 2,000 IU/day over a period of 12 weeks and afterwards had their 25(OH)D level determined. Vitamin D insufficiency and deficiency (<75 mmol/L and <30 ng/mL, respectively) was diagnosed in 78.0 and 61.0% of the patients, respectively. After the 12-week therapy, calcifediol concentrations were normalized in 89.0% of healthy subjects and only in 50.0% of obese adolescents. In their conclusion, the investigators recommend a dose increase for adolescents with obesity (111).

In another trial conducted in 68 obese adolescents (median BMI: 38.0 kg/m^2^), administration of vitamin D 50,000 IIU once weekly for a period of 6–8 weeks allowed normalization of 25(OH)D concentrations only in 28.0% of the adolescents, while a repeated course of the same duration and at the same dose level produced no significant changes in the remaining 72.0% (112).

A study that was conducted in the Russian Federation (Saint Petersburg) by I. L. Nikitina, which enrolled children with obesity (median BMI: 29.6 kg/m^2^), revealed low vitamin D concentrations (<75 nmol/L, <30 ng/mL) in 92.0% of subjects. Supplementation with cholecalciferol 1,500 IU/day for 3 months, followed by 2,000 IU/day for 3 months, helped normalize calcifediol concentrations in 41.0% of the children. Calcifediol levels returned to normal within the first 3 months, and subsequent supplementation at a higher dose over the same period of time did not change the vitamin D insufficiency rate (80).

## Summary and Conclusion

The high rates of excessive body weight and obesity observed worldwide and vitamin D insufficiency are closely interrelated problems of today's medicine that indicate a pandemic and have deleterious health effects in large patient populations.

Excessive body weight results in accumulation of adipose tissue, impaired adipocyte function, development of adipocyte hypertrophy, and an altered adipokine secretion profile. These changes result in migration and transformation of macrophages and in the development of adipose tissue inflammation. As a result of this inflammation, the synthesis of pro-inflammatory cytokines (TNF-alpha, IL-6, and IL-1β) becomes increased and insulin resistance develops.

Vitamin D has a modulatory effect on the expression of the genes responsible for secretion of leptin and adiponectin. *In vitro*, 25(OH)D metabolites inhibit chronic immune-mediated inflammation by suppressing the production of the pro-inflammatory cytokines IL-1β, IL-6, and IL-8.

Long-term monitoring of obese patients receiving vitamin D supplementation revealed an improvement of the adipose tissue inflammation that was a result of inhibited TNF-alpha activity. Vitamin D supplementation in patients with diabetes mellitus type 2 helps decrease C-reactive protein and TNF-alpha concentrations, decrease ESR, and increase leptin concentrations.

The prevalence of vitamin D insufficiency among children and adolescents with obesity is extremely high: 96.0% in Germany, 78.4% in the United States, and up to 92.0% in the Russian Federation.

Despite the consensus achieved with regard to the need to treat vitamin D insufficiency in obese patients, there is no common point of view on the dosage and duration of cholecalciferol administration appropriate for vitamin D supplementation. Currently available data on the treatment of vitamin D insufficiency in obese children and adolescents are contradictory; however, in the overwhelming majority of cases these data allow not only an increase in calcifediol levels but also a positive effect on carbohydrate and lipid metabolism, as well as on the secretion of adipokines.

## Author Contributions

IZ: chief of our group; LK: the mechanism of action of vitamin D in obesity; VK: effects of vitamin D on congenital and acquired immune response; IN: prevalence of vitamin D insufficiency in overweight and obese children and adolescents, vitamin D doses in overweight and obese children; SM: prevalence of vitamin D insufficiency in overweight and obese children and adolescents, vitamin D doses in overweight and obese children; SD: prevalence of vitamin D insufficiency in overweight and obese children and adolescents; AK: adipose tissue and its effects on autoimmune inflammation; RA: prevalence of vitamin D insufficiency in overweight and obese children and adolescents; MS: interrelationship between vitamin D and adipose tissue; AT: adipose tissue and autoimmune inflammation; GK: effects of vitamin D on adipokine levels and metabolic specifics in overweight and obese children; AL: prevalence of overweight and obesity in children and adolescents.

### Conflict of Interest Statement

The authors declare that the research was conducted in the absence of any commercial or financial relationships that could be construed as a potential conflict of interest.
